# Global perspectives from women leaders in radiology: a view from five continents

**DOI:** 10.1093/bjr/tqaf259

**Published:** 2025-10-22

**Authors:** G McGinty, E L M Ho, C Jones, L Leong, A Rockall, N Touil, B E González Ulloa

**Affiliations:** Weill Cornell Medicine Departments of Radiology and Population Health Sciences, New York, NY 10022, United States; Imaging Department, ParkCity Medical Centre, Kuala Lumpur, 52200, Malaysia; Department of Medical Imaging, Royal Brisbane and Women’s Hospital, Herston, Brisbane, 4006, Australia; Faculty of Medicine and Health, University of Sydney, Sydney, 2000, Australia; Hong Kong College of Radiologists , Hong Kong SAR, China; Department of Surgery and Cancer, Faculty of Medicine, Imperial College London, London W12 0NN, United Kingdom; Department of Emergency Radiology, Hassan II University of Casablanca, 20360, Morocco; Breast Imaging Department, Diagnostico Especializado por Imagen, Zapopan, Jalisco, 45040, Mexico

**Keywords:** leadership, women, global, radiology

## Abstract

Women are not a monolithic group and healthcare systems vary widely across the world. Nonetheless there are lessons to be learned from the experience of women who have achieved leadership roles no matter where or in what field. Emanating from Asia, Australia, the Americas, Northern Europe and Africa, the authors’ perspectives on the barriers and opportunities for women seeking advancement in radiology offer both a guide and a call to action. The authors include current and past leaders of national and continental radiology organizations. Several were the first woman to hold the position. The participation of women in the highest levels of healthcare and policy leadership continues to lag that of men yet studies demonstrate that when women lead, the quality of care and patient satisfaction improve.

Women are not a monolithic group and healthcare systems vary widely across the world. Nonetheless there are lessons to be learned from the experience of women who have achieved leadership roles no matter where or in what field. Emanating from Asia, Australia, North America, Northern Europe and Africa, the authors’ perspectives on the barriers and opportunities for women seeking advancement in radiology offer both a guide and a call to action.

The authors include current and past leaders of national and continental radiology organizations. Several were the first woman to hold the position. The participation of women in the highest levels of healthcare and policy leadership continues to lag that of men yet studies demonstrate that when women lead, the quality of care and patient satisfaction improve.^1^^,^^2^

## Background on health systems

The Australian health care system is made up of both public hospital medicine and a strong private sector. In the diagnostic imaging sector, the majority of radiology investigations are performed by private radiology providers. Women comprise the majority of medical school graduates in Australia, however only 30% of radiologists are women with practice ownership even more skewed toward men.

Hong Kong SAR, China has a hybrid system for healthcare delivery. 90% of the in-patients are under care of public hospitals whereas for out-patients, 90% of care is provided by private practice physicians. 80% of healthcare providers in Hong Kong are women, 72.4% in China.^3^ Again, despite these numbers, women are underrepresented in leadership roles.^4^ The percentage of women in the radiology workforce in Hong Kong SAR is 37%.

Malaysia also has a dual healthcare system with the Ministry of Health providing universal healthcare services funded by taxes to its citizens and legal residents. The parallel private healthcare service is funded by out-of-pocket payments or private health insurance.^6^ In 2022, 61% of the students enrolled in Malaysian public universities were female.^6^ About 60% of registered Malaysian radiologists are women. 50% of all radiologists work in the public system and in the last 20 years there have been four consecutive female heads of radiologic services for the entire public health system.^6^

Mexico’s healthcare system is a hybrid model, composed of public and private sectors. The public sector serves about 65% of the population and includes social security institutions which provide care based on formal employment, and the Ministry of Health (SSA), which serves the uninsured population. The private sector provides care to about 30%, operates independently and caters mostly to middle- and upper-income groups. Those seeking private sector care often do so to supplement public services when access or quality is perceived to be lacking. Women account for over 50% of medical school graduates and a growing percentage of practicing physicians. However, they remain a minority at approximately 43% in radiology^7^ and are underrepresented in senior leadership positions such as hospital directors, deans, heads of specialty departments, and national boards.

Morocco’s healthcare system is divided between a public sector—which serves the majority but is weakened by regional disparities and limited resources—and a private sector, which is more efficient but often accessible only to wealthier segments of the population. Although women make up 58% of healthcare professionals, they struggle to reach leadership positions.

The United Kingdom’s (UK) National Health Service is predominantly provided by the publicly funded NHS, through taxation. Private practice is limited but more common in large urban centers. Entrance to medical school is now 60% female and as of 2025, there are more women on the medical register with a license to practice than men but women only make up 37% of the radiology workforce.^8^

The United States of America’s health system is a public/private hybrid. Government programs cover the poor and the elderly but most obtain health insurance through their employer. While entrance to medical school is approximately 50% female, the radiology workforce in North America is less than a third female and the percentage drops precipitously for leadership roles.^5^

## Barriers

Barriers to women entering the radiology workforce are both perceived and real. Myths about the fear of radiation and distaste for physics^9^ are balanced by the reality of a long training period and, in countries such as Australia, the expectation of doing regional and remote rotations at a time when many women are starting their family. While private practice is typically more lucrative, benefits such as flexible working hours and parental leave may be more codified for public healthcare positions.

Negativity around women taking parental leave leading to reluctance to recruit women appears to be universal whether it’s the UK’s generous 1 year leave or the US’s mere weeks. More affordable child care in Malaysia contrasts with a fragmented and prohibitively expensive system in the US. The gender pay gap varies and may evade equal opportunity laws if related to men’s greater opportunities to add private practice income to their public health system salary as in the UK. Certain in-demand specialties in the US such as breast imaging have, however, recently commanded higher salaries for the majority female workforce in these disciplines.

Cultural expectations and gender stereotypes persist. Expectations that women will be responsible for the majority of domestic responsibilities may be more or less explicit but are widespread. Intersectional barriers such as race, age and religious affiliations also play a significant role in the lower participation of women in leadership.

While none of the authors practice in regions with structural barriers to women’s participation in leadership roles, the existence of a “glass ceiling” was broadly acknowledged especially in certain sectors such as the more lucrative private practice in the UK, US and Australia. Decision-making in private practice groups may be less subject to scrutiny and allow implicit bias against women leaders to persist.

Attendance at conferences and involvement in organized radiology is a universal part of the path to leadership. The emergence of hybrid meetings affords women more opportunities to attend but may not provide them the same advancement credits as in person participation. The US’s premier scientific meeting, that of the Radiologic Society of North America, has provided childcare facilities for many years but most conferences still lack childcare or lactation facilities. Much of the valuable networking happens in after-hours settings that are not designed for those with young families. Mentorship and leadership development programs tailored to the specific needs and realities of women are often lacking.

A “cultural tax”^10^ has been described that minority physicians pay through their perceived obligation to mentor minority students and trainees as well as take on leadership roles related to diversity. These responsibilities are often uncompensated, and unrecognized, and some roles may result in pushback from policy makers if their research and activities reveal deficiencies in organizations. Taking on these additional obligations may conflict with their own professional leadership advancement and may lead to moral distress.^11^

The business literature has documented extensively the ways in which women’s ideas and voices are less likely to be heard. Evidence also abounds that women are less likely to receive grant funding, publish first author papers and receive honors and awards. These biases do seem to have a wide global reach.

The lack of female role models in leadership is a variable barrier. There has not been a female clinical radiologist as president of RANZCR since 1985^12^ but across Asia many women have held leadership roles in professional societies. Women have led all major US professional radiology societies and the European Society has had multiple female leaders as has the Royal College of Radiologists.

## Opportunities

Some countries, for example, Mexico have adopted legislative and institutional reforms that mandate gender parity in some public sector appointments, opening doors for women to serve on hospital boards and regulatory bodies. Morocco has implemented reforms to promote equality, such as the National Strategy for Gender Equality (2015-2030).^13^ Others, the US for example, have not yet codified equal rights for women which affords an opportunity for positive change through legislative and policy advocacy.

Professional organizations such as the United States’ American Association for Women in Radiology are focused on providing networking and advocacy for the advancement of women in radiology. These organizations provide opportunities for community building and a platform for influencing policy on topics such as parental leave, flexible and hybrid working and pay equity. While not every country has a dedicated radiology professional organization for women, many have initiated committees focused on diversity such as the Royal College of Radiologists’ Equity Diversity and Inclusion Committee. Recognition of the gender disparity in organizational leadership in Australia has led to the formation of multiple groups and societies in different states across Australia to focus on women in radiology leadership including the Royal Australian and New Zealand College of Radiologists’ Diversity, Equity and Inclusion taskforce.

The ability to network within international radiology through attendance at conferences and participation on collaborative projects offers additional platforms for women in radiology to engage with global peers and assume visible leadership roles.

Seeking roles outside of one’s home institution or professional society can afford women additional opportunities to lead. Participation in government working groups or initiatives or membership of public and non-profit boards can be an important place to hone leadership and governance skills. These skills are eminently transferrable to the workplace.

The importance of role models of women leaders is believed by all authors to offer an important catalyst for more diverse leadership. This is compounded by the benefits conferred by the availability of women leaders as mentors and sponsors. For those role models to have had careers that included periods of working part time due to family commitments or have had career pivots is especially powerful.

While leadership development programs focused on the career challenges and opportunities faced by women are not universally available, they are increasing in frequency. While there are local norms and structures that must inform content, the principles are quite transferable which provides ample opportunity to scale programs at low cost.

Asked for their tips for those women who seek leadership roles, the authors offer the following:

## Ten top tips from global women leaders in radiology for women seeking to lead

1. Articulate your values and hold to them, you are unlikely to be successful in an organization whose values diverge widely from yours.
2. Empathy and kindness may be perceived as traditionally female leadership traits and as negatives but will drive organizational excellence.
3. Lead from where you are. Influence can often be more effective than authority.
4. Build your network and ask for help when you need it. Seek diverse mentors and sponsors.
5. Set clear goals. Develop and maintain a strong personal brand but don’t box yourself in to one path.
6. Build as much flexibility as possible to allow you to seize opportunities when they present themselves. Stretch out of your comfort zone and don’t shy away from challenging roles but beware the “glass cliff” that often plagues women leaders who are only offered roles at a time of inevitable organizational decline.
7. Be strategic with your time: Prioritize opportunities that align with your goals and make your impact visible. Take care of yourself.
8. Actively listen but then also have the confidence to express your own opinions or ideas in meetings. Own your expertise: Speak with authority. Your training and results are your strongest credentials. Find the framing that makes your position stronger. Challenge bias directly and professionally: Don’t be afraid to point out inequality. Change often begins by naming the problem.
9. Ask for the position you want: men do this more than women
10. Recognize your opportunity to drive change as a leader and use that for good. Support other women: Leadership is not a solo journey. Open doors for those who come after you.

## Conclusion

Across the world, health systems face both challenges and opportunities. The workforce challenge in radiology is global as is the promise and potential of artificial intelligence. Aging populations in Asia, US and the UK contrast Africa’s young and growing population. A sustainable future for radiology and for healthcare requires leadership that leverages the full talent and diversity of the workforce. Women have already proven their capability at every level of care. It is time to ensure we are equally represented where decisions are made.
